# Investigation of Four Cases of Stevens–Johnson Syndrome among Participants in a Mass Drug Administration Campaign with Sulfadoxine-Pyrimethamine and Primaquine in Haiti, 2020

**DOI:** 10.4269/ajtmh.22-0625

**Published:** 2023-05-01

**Authors:** Michelle A. Chang, Bernadette Fouché, Willy LaFortune, Kathleen Holmes, Jonas Rigodon, Stanley Juin, Samson Marseille, Eric Rogier, Michael Green, Taba Kheradmand, Samuel G. Moore, David A. Gaul, Jacques Boncy, Marc-Aurele Telfort

**Affiliations:** ^1^ Malaria Branch, Division of Parasitic Diseases and Malaria, Center for Global Health, Centers for Disease Control and Prevention, Atlanta, Georgia;; ^2^ CDC Foundation, Atlanta, Georgia;; ^3^ Programme National de Contrôle de la Malaria, Ministère de la Santé Publique et de la Population, Port-au-Prince, Haiti;; ^4^ CDC-Haiti, Division of Global Health Protection, Center for Global Health, Centers for Disease Control and Prevention, Port-au-Prince, Haiti;; ^5^ Laboratoire National de la Santé Publique, Ministère de la Santé Publique et de la Population, Port-au-Prince, Haiti;; ^6^ Entomology Branch, Division of Parasitic Diseases and Malaria, Center for Global Health, Centers for Disease Control and Prevention, Atlanta, Georgia;; ^7^ Fred H. Allen Immunogenetics Laboratory, New York Blood Center, New York, New York;; ^8^ Parker H. Petit Institute for Bioengineering and Bioscience, Georgia Institute of Technology, Atlanta, Georgia;; ^9^ School of Chemistry and Biochemistry, Georgia Institute of Technology, Atlanta, Georgia

## Abstract

In 2018, a mass drug administration (MDA) campaign for malaria elimination was piloted in Haiti. The pilot treated 36,338 people with sulfadoxine-pyrimethamine (SP) and primaquine; no severe adverse events were detected. In 2020, another MDA campaign using the same medications was implemented to mitigate an upsurge in malaria cases during the COVID-19 pandemic. Four cases of Stevens–Johnson syndrome (SJS) were identified among the 42,249 people who took the medications. Three of these individuals required hospitalization; all survived. In addition to SP ingestion, an investigation of potential causes for increased SJS cases identified that all four cases had human leukocyte antigens A*29 and/or B*44:03, another known risk factor for SJS. Additionally, three of the four case individuals had antibodies to SARS-CoV-2, and the fourth may have been exposed around the same time. These findings raise the possibility that recent SARS-CoV-2 infection may have contributed to the increased risk for SJS associated with SP exposure during the 2020 campaign.

The Ministry of Public Health and Population in Haiti (MSPP; French acronym) strives to eliminate malaria by 2025. The number of presumed and confirmed *Plasmodium falciparum* malaria cases decreased from 34,350 in 2011 to 8,828 in 2018, and then rose to 22,987 in 2020.^1^ A pilot mass drug administration (MDA) and indoor residual spraying campaign was conducted in communities with malaria transmission foci in the department of Grand’Anse during October–November 2018.^2^ The pilot treated 36,338 people with a one-dose regimen of sulfadoxine-pyrimethamine (SP) using weight-associated age categories according to Haiti’s national policy and a single low dose of primaquine (SLD PQ) for the gametocytocidal effect as recommended by the WHO.^3^ Both medications had been in use in Haiti prior to the campaigns.^4^ In 2018, no severe adverse events (SAEs) associated with the MDA campaign were detected by pharmacovigilance (PV).

The MSPP implemented an MDA-only campaign in 2020 using the same medications, doses, and monitoring procedures as in 2018. The 2020 campaign aimed to mitigate an upsurge of malaria cases that was likely due to disrupted health services caused by political unrest and the COVID-19 pandemic. Passive PV was implemented to detect SAEs such as Stevens–Johnson syndrome (SJS), which has been associated with SP ingestion.^5^ Most frequently associated with medications, SJS is a rare and potentially fatal immune-mediated reaction that appears as a blistering skin rash and mucosal ulcerations.^6^

The 2020 campaign began on September 23 in Grand’Anse. By October 28, three cases of SJS (two took SP and SLD PQ; one took SP only) requiring hospitalization were reported. Because the number of SJS cases exceeded expectations, the campaign was halted on October 28, at which time 42,249 people had been treated (90% SP and SLD PQ; 10% SP only). Active surveillance was initiated for additional SJS cases. Passive PV continued for 30 days, during which time a fourth individual with SJS was identified (Table 1).

**Table 1:** Clinical histories of case individuals with Stevens–Johnson syndrome after a mass drug administration campaign, Haiti, October–November 2020

Case individual	Age (years), sex	Weight at the time of evaluation for SJS (kg)	Clinical course based on patient interviews, campaign data, and clinical reports
1-0	12, M	27	10/5: ingested SP 1,250/62.5 mg and PQ 11.2 mg.
			10/16: symptoms started with pruritus of the lips and face followed by the appearance of vesiculopapular lesions that became excoriated, and a fever developed; the rash was complicated by ulceration of the lips and oral cavity associated with throat swelling and breathing difficulties.
			10/24–11/7: admitted to a reference hospital; hospital course was uncomplicated; discharged in good condition.
2-0	15, F	43	10/1: ingested SP 1,500/75 mg and PQ 15 mg.
			10/18: symptoms started with generalized, eruptive cutaneous lesions, then ulcers appeared on the lips and oral cavity; lesions became superinfected; difficulty in breathing developed with overall deterioration.
			10/23–11/7: admitted to a reference hospital; hospital course was uncomplicated; discharged in good condition.
3-0	34, F	65	10/7: ingested SP 1,500/75 mg.
			10/7: within 24 hours symptoms started with pruritis of the tongue and lips; after 2 days, hives with skin edema (peau d’orange) developed; a home remedy of an herbal poultice was applied to the skin that aggravated the rash; ulcerations began in the mouth and lips.
			10/27: admitted to a reference hospital appearing disoriented and septic; hospital course complicated by eye pain and eyelids sticking together requiring ophthalmologic treatment; clinically stable for discharge by 11/16. Postdischarge course was complicated by the development of corneal ulcers requiring evaluation and treatment by an ophthalmologist over the following 3 months.
4-0	50, F	68	10/1: ingested SP 1,500/75 mg and PQ 15 mg.
			10/21: symptoms began with vesicles on the lips, inflammation of the face, and dysphagia for solids; excoriated papular lesions developed on the chest and left arm; the rash was complicated by a skin ulcer.
			10/5 (estimated): sought outpatient treatment at a local health center; by 11/1 overall improvement with lip ulcers and skin lesions healing.

F = female; M = male; PQ = primaquine; SJS = Stevens–Johnson syndrome; SP = sulfadoxine-pyrimethamine. SP: target dose of 25/1.25–33/1.7 mg/kg; maximum 1,500/75 mg; PQ: target dose of 0.25 mg/kg; maximum 15 mg.

Although estimates vary for the occurrence of SJS or any SAE associated with a sulfonamide depending on the population, the study methods, and the context of the medication exposure, four cases of SJS among 42,249 people exceeded the expected rate reported in the published literature and from the previous pilot campaign.^5^^,^^7^^–^^9^ One relevant study from Malawi found 1.7 severe cutaneous adverse reactions in 100,000 SP exposures observed over 18 months for individuals aged ≥ 15 years.^10^

In addition to medications, infections (e.g., mycoplasma pneumoniae, HIV), medical conditions (e.g., malignancy), herbal remedy use, and some human leukocyte antigen (HLA) genotypes have been associated with SJS.^6^ Recently, multiple case reports described SJS in patients with COVID-19 and exposure to medications, including hydroxychloroquine.^11^^–^^13^ The main difference between the 2018 pilot campaign context and that of 2020 was the appearance of the COVID-19 pandemic. The change in context raised the possibility that SARS-CoV-2 infection might have increased the risk for SJS. Additionally, the use of other medications such as chloroquine (CQ) or herbal remedies to prevent or treat COVID-19 has been documented and warranted investigation as possible contributors to the number of SJS cases.^14^^–^^16^

To investigate potential risk factors, the MSPP team interviewed case individuals for history of medication allergies, recent illnesses, malignancy, HIV infection, recent medication or herbal remedy ingestion, and the clinical course of SJS symptoms. Data from campaign monitoring were used to corroborate the dates, medications, and doses of SP and PQ exposures. We tested by high-performance liquid chromatography the percentage of active pharmaceutical ingredients (APIs) of all lots of SP and PQ deployed in the campaign. Approximately 6 weeks after ingesting the MDA medications, nasopharyngeal swabs and blood samples were collected from three case individuals and their household members during November 17–20, 2020; samples were tested for SARS-CoV-2 DNA by polymerase chain reaction and antibodies by multiplex bead assay (Tetracore, Inc., Rockville, MD). In addition, all dried blood samples from November were analyzed with targeted ultra-performance liquid chromatography-mass spectrometry (UPLC-MS) to quantitate SP, PQ, CQ, and their metabolites (see Table 2 for limits of detection). Blood tests for only the case individuals were completed 6 months after the first samples for HLA genotyping; this second visit provided the opportunity for follow-up testing for SARS-CoV-2 antibodies for all four case individuals. Limited resources prevented conducting HLA genotyping on all household members. The HLA genotyping for HLA-A, B, C, DRB, DQA/DQB, and DPA/DPB alleles was performed by next-generation sequencing hybrid capture assay and analyzed using AlloSeq Assign software (CareDx, Brisbane, CA). The small number of cases precluded a case–control analysis.

**Table 2:** Possible risk factors for Stevens–Johnson syndrome in case individuals and family members, Haiti, October 2020–June 2021

Individual code*	Age (years)	Sex	MDA†	HLA genotype	SARS-CoV-2 PCR (November 17–20, 2020)	SARS-CoV-2 IgG no. 1 (November 17–20, 2020)	SARS-CoV-2 IgG no. 2 (May 31, 2021)	Concentration of medication metabolites detected in blood samples (nM) (November 17–20, 2020)‡§	
								Sulfadoxine	Chloroquine
1-0	12	M	SP, PQ	B*44:03, heterozygous	ND	Pos	Equivocal	60.2	ND
1-1	16	M	SP, PQ	–	ND	Pos	–	235.4	35.9
1-2	15	F	SP, PQ	–	ND	Pos	–	159.8	ND
1-3	9	F	SP, PQ	–	ND	Neg	–	417.5	ND
1-4	41	M	SP, PQ	–	–	–	–	–	–
1-5	35	F	Excluded	–	–	–	–	–	–
1-6	13	M	SP, PQ	–	–	–	–	–	–
1-7	56	M	SP, PQ	–	–	–	–	–	–
2-0	15	F	SP, PQ	B*44:03, heterozygous	ND	Pos	Equivocal	338.7	ND
2-1	52	F	SP, PQ	–	ND	Pos	–	906.8	ND
2-2	10	F	SP, PQ	–	ND	Pos	–	45.7	ND
2-3	9	M	SP, PQ	–	ND	Pos	–	12.8	5289.7
2-4	32	F	SP, PQ	–	ND	Pos	–	ND	ND
2-5	34	F	SP	–	–	–	–	–	–
2-6	30	M	Refused	–	–	–	–	–	–
2-7	0	F	Excluded	–	–	–	–	–	–
3-0	34	F	SP	A*29, heterozygous B*44:03, homozygous	ND	Pos	Equivocal	201.1	ND
3-1	45	M	SP, PQ	–	ND	–	–	719.7	ND
3-2	12	F	SP, PQ	–	–	Neg	–	728.8	ND
3-3	10	F	SP, PQ	–	–	Neg	–	413.1	ND
3-4	9	F	SP, PQ	–	ND	Neg	–	28.6	ND
3-5	7	M	SP, PQ	–	–	Neg	–	87.5	ND
3-6	4	M	SP, PQ	–	ND	Neg	–	247.3	ND
3-7	1	F	SP	–	–	Neg	–	113.7	ND
3-8	–	–	Yes	–	–	–	–	–	–
4-0	50	F	SP, PQ	B*44:03, heterozygous	–	–	Equivocal	–	–
4-1	48	M	SP, PQ	–	–	Pos	–	254.6	ND
4-2	7	F	SP, PQ	–	–	Pos	–	82.7	ND
4-3	–	–	Yes	–	–	Neg	–	64.9	ND
4-4	13	M	SP, PQ	–	–	–	–	–	–
4-5	20	F	SP, PQ	–	–	–	–	–	–
4-6	17	M	SP, PQ	–	–	–	–	–	–
4-7	21	M	SP, PQ	–	–	–	–	–	–
4-8	11	M	SP, PQ	–	–	–	–	–	–
4-9	24	M	SP, PQ	–	–	–	–	–	–
4-10	28	M	SP, PQ	–	–	–	–	–	–

F = female; HLA = human leukocyte antigen; M = male; MDA = mass drug administration; ND = not detected; Neg = negative; PCR = polymerase chain reaction; Pos = positive; PQ = primaquine; SP = sulfadoxine-pyrimethamine. Dashes indicate that results were not available because of resource limitations (HLA genotype testing and SARS-CoV-2 IgG in May 2021 were only conducted for case individuals); for all other assays, teams attempted to collect blood and nasopharyngeal samples from all case individuals and household members; here, dashes indicate that results were unavailable because of individual unavailability or refusals.

* Individual code designates the household by 1, 2, 3, and 4 as the first digit; the second digit with “-0” indicates the case individual; all others are noncase household members.

† “SP, PQ” or “SP” indicates the medication was confirmed as taken in the MDA campaign; “yes” indicates a personal report of taking MDA medications; MDA occurred on October 1–7, 2020, for these households.

‡ Ultra-performance liquid chromatography-mass spectrometry (UPLC-MS) concentration limits of detection: sulfadoxine: 3.4 nM; chloroquine: 35.1 nM; primaquine: 3.7 nM. Primaquine was not detected in any of the same samples that were tested for sulfadoxine and chloroquine; primaquine results are not shown.

§ UPLC-MS analysis of the same samples did not detect any chemical compounds for the following plants used as herbal remedies (local names): *Artemesia vulgaris* (armoise), *Azadirachta indica* (lila), and *Momordica charantia* (asorossi).

Consent for interviews and blood collection was conducted as per the routine procedures of the MSPP. Written consent for HLA genotyping at the New York Blood Center was provided from the four case individuals. The investigation protocol was reviewed by the CDC and was conducted consistent with applicable federal law and CDC policy (see e.g., 45 C.F.R. part 46, 21 C.F.R. part 56; 42 U.S.C. §241(d); 5 U.S.C. §552a; 44 U.S.C. §3501 et seq).

The % API of the SP and PQ lots were within acceptable range. The interviews confirmed that the four case individuals had no prior medical conditions or drug allergies. None had participated in the 2018 MDA pilot. Only case 4-0 reported taking any medication (paracetamol) within 2 weeks of the MDA; all reported consuming herbal or traditional remedies during April–May to prevent COVID-19. Reviews of SP and PQ doses administered to the case individuals were accurate according to the age-based categories. However, for the two youngest (cases 1-0 and 2-0), the doses were slightly higher than the target when calculated using their weights at the time of their clinical evaluation for SJS (Table 1). Among the four cases, case 3-0 took only SP because she was breastfeeding at that time. This finding, plus the fact that PQ has not been associated with SJS, suggested that PQ was likely not a contributing factor. No other household members experienced symptoms of SJS and almost all (90.6%) took SP with or without PQ (Table 2).

The UPLC-MS analysis identified the metabolites of SP as expected; however, no metabolites for PQ were detected. This finding corresponded with the very low dose of PQ administered for its gametocytocidal effect against *Plasmodium* parasites. Although the MDA campaign excluded individuals who reported taking CQ or other antimalarials, we detected CQ metabolites in the November blood samples from two noncase individuals from different households. This finding suggested that concomitant CQ use was likely not a risk factor for the case individuals. No specific metabolites associated with commonly used plants were observed by nontargeted UPLC-MS analysis of any blood samples from case individuals or their household members to support individual consumption.

Results for HLA genotypes for case 3-0 revealed the presence of A*29 (heterozygous) and B*44:03 (homozygous); the remaining three case individuals were heterozygous for B*44:03. In an early European study of severe SJS, the combination of A*29 and B*12 (of which B*44:03 is a subgroup) was associated with a 13.4% increased relative risk with sulfonamide exposure when compared with controls (*P* corrected < 0.04).^17^ Other studies have shown that HLA-B*44:03 is associated with SJS with severe ocular complications with the consumption of common cold medicines in Indian, Brazilian, and other populations.^18^^,^^19^ Case 3-0 had both A*29 (heterozygous) and B*44:03 (homozygous) genotypes, and experienced the most severe symptoms with ocular complications (Figure 1). In the United States, the frequency of the A*29 and B*44:03 alleles each is approximately 7% and 10% among different ethnic populations and 3.7% and 5.4% within persons of African descent.^20^ The frequency of such alleles occurring together is close to 1% in those of African descent. Although it is difficult to interpret the HLA results without data on the genotype prevalence in Haiti, the genotype prevalence was likely the same during both MDA campaigns. In this context, the HLA findings suggested that the genotypes may have increased the susceptibility for the case individuals; however, there was an additional factor in 2020 not present in 2018.

**Figure 1.**
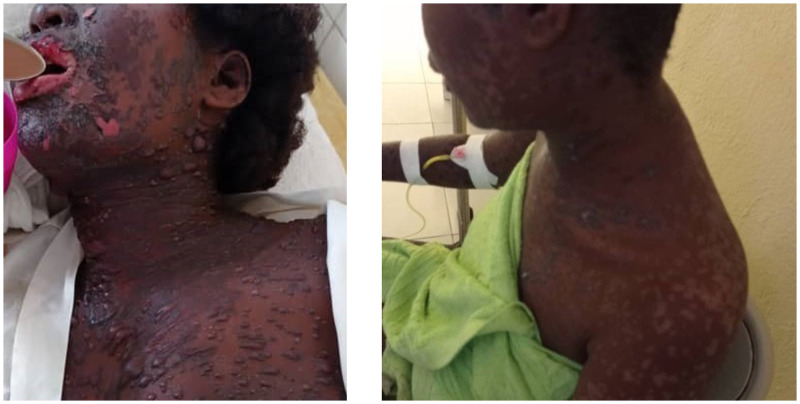
Case 3-0 on October 28 (left) with severe Stevens–Johnson syndrome associated with sulfadoxine-pyrimethamine ingestion and human leukocyte antigen genotypes A*29 and B*44:03, and then with improvement by November 12 during hospitalization (right).

Although no SARS-CoV-2 was detected in any nasopharyngeal sample, antibody results available for three case individuals suggested that they had been exposed to SARS-CoV-2 before November 2020, when samples were collected (6 weeks after the MDA). However, it is not possible to determine precisely when the acute infection occurred. The equivocal follow-up antibody results in May 2021 were consistent with waning antibody levels and supported the hypothesis that case 4-0, for whom there was no earlier blood sample, was possibly infected at the same time as the others in November. Two household members of case 4-0 had antibodies in November, which indicated that household exposure to SARS-CoV-2 occurred prior to November 2020. These data raise the possibility that SARS-CoV-2 infection could have contributed to the risk of SJS if the acute infection occurred at the time of the MDA.

Our investigation identified several risk factors for SJS among the four case individuals. Based on our pilot in 2018 and global experience, it is unlikely that a single exposure to SP for malaria treatment was the sole cause of all four SJS cases in 2020. Three of the four case individuals had definitive evidence of SARS-CoV-2 infection prior to November 2020, based on the presence of antibodies, and carried at least one HLA marker for genetic susceptibility to SJS. The only new factor in 2020 that was identified was the appearance of SARS-CoV-2. Although SP has been used safely in Haiti, the introduction of a widespread, novel infection may have contributed to the increased risk of SJS with SP exposure during the 2020 MDA campaign. In Haiti, we discourage the use of SP for MDA in the presence of circulating SARS-CoV-2 until additional evidence is available on the potential interaction with the novel virus, especially among individuals with certain HLA genotypes. Elsewhere, prioritizing PV in programs that use SP in population-based chemoprevention strategies (i.e., seasonal malaria chemoprophylaxis; intermittent prevention and treatment in pregnancy) may aid in understanding the potential risk of SJS related to SP and SARS-CoV-2.

## Financial Disclosure

Funding was provided by the US Centers for Disease Control and Prevention and by a grant from the Bill & Melinda Gates Foundation (OPP1114297) to the CDC Foundation as part of the Malaria Zero Consortium (https://www.cdcfoundation.org/). Funding for the ultra-performance liquid chromatography-mass spectrometry was provided by the Bill & Melinda Gates Foundation through the Clinton Health Access Initiative.
